# Genomic analysis of *Helicobacter himalayensis* sp. nov. isolated from *Marmota himalayana*

**DOI:** 10.1186/s12864-020-07245-y

**Published:** 2020-11-23

**Authors:** Shoukui Hu, Lina Niu, Lei Wu, Xiaoxue Zhu, Yu Cai, Dong Jin, Linlin Yan, Fan Zhao

**Affiliations:** 1 Department of Clinical Laboratory, Peking University Shougang Hospital, Beijing, 100144 China; 2 Department of Pathogen Biology, School of Basic Medicine and Lifescience, Hainan Medical University, Haikou, 571101 China; 3 State Key Laboratory of Infectious Disease Prevention and Control, Collaborative Innovation Center for Diagnosis and Treatment of Infectious Diseases, National Institute for Communicable Disease Control and Prevention, Chinese Center for Disease Control and Prevention, Changping, Beijing, 102206 China

**Keywords:** Helicobacter, Comparative genomics, *Helicobacter himalayensis*, Virulence factor

## Abstract

**Background:**

*Helicobacter himalayensis* was isolated from *Marmota himalayana* in the Qinghai-Tibet Plateau, China, and is a new non-*H. pylori* species, with unclear taxonomy, phylogeny, and pathogenicity*.*

**Results:**

A comparative genomic analysis was performed between the *H. himalayensis* type strain 80(YS1)^T^ and other the genomes of *Helicobacter* species present in the National Center for Biotechnology Information (NCBI) database to explore the molecular evolution and potential pathogenicity of *H. himalayensis*. *H. himalayensis* 80(YS1)^T^ formed a clade with *H. cinaedi* and *H. hepaticus* that was phylogenetically distant from *H. pylori*. The *H. himalayensis* genome showed extensive collinearity with *H. hepaticus* and *H. cinaedi*. However, it also revealed a low degree of genome collinearity with *H. pylori*. The genome of 80(YS1)^T^ comprised 1,829,936 bp, with a 39.89% GC content, a predicted genomic island, and 1769 genes. Comparatively, *H. himalayensis* has more genes for functions in “cell wall/membrane/envelope biogenesis” and “coenzyme transport and metabolism” sub-branches than the other compared helicobacters, and its genome contained 42 virulence factors genes, including that encoding cytolethal distending toxin (CDT).

**Conclusions:**

We characterized the *H. himalayensis* 80(YS1)^T^ genome, its phylogenetic position, and its potential pathogenicity. However, further understanding of the pathogenesis of this potentially pathogenic bacterium is required, which might help to manage *H. himalayensis*-induced diseases.

**Supplementary Information:**

The online version contains supplementary material available at 10.1186/s12864-020-07245-y.

## Background

The *Helicobacter* genus comprises gram-negative bacteria with characteristic spiral shapes [1]. The type species, *Helicobacter pylori*, was the first cultivated species of the genus. *H. pylori* was first isolated from in the stomachs of patients with gastritis and peptic ulceration in 1984 [2]. Currently, over 30 species have been isolated, including many candidate or unclassified species [3]. Except for *H. pylori*, most of the other *Helicobacter* have been isolated and identified from wild animals, including rodents, cats, dogs, rabbits, chickens, sheep, cattle, swine, cheetahs, ferrets, dolphins, whales, and non-human primates [4–8]. In addition, certain Helicobacters were isolated from laboratory animals [7, 9–11]. Convincing evidence has identified *H. pylori* as a causative bacterium of gastric adenocarcinoma, mucosa-associated lymphoid tissue lymphoma, and peptic ulcer in humans [12]. However, studies of the association between diseases and non-*H. pylori* species are lacking. There are only a few reports about non-*H. pylori* infection in humans, mostly in immunocompromised patients [13–16]. Thus, the pathogenicity and genetic characteristics of non*-H. pylori* remain to be explored.

In 2015, we reported the isolation of *Helicobacter himalayensis sp.* nov. strain 80(YS1)^T^ from *Marmota himalayana* in the Qinghai-Tibet Plateau, China. This was the first report that a new species of helicobacter could be isolated and cultured directly in the gastrointestinal mucosa of a *Marmota himalayana. M. himalayana* live in the Qinghai-Tibet Plateau, a relatively isolated environment with an average altitude over 4000 m, which induces great differences in animal and plant biodiversity compared with plain regions. *H. himalayensis*, a new species of *Helicobacter* isolated from this plateau, may have unique evolutionary, pathogenic, and drug resistance characteristics because of its unique natural environment and host.

With development of next-generation sequencing technologies, many whole-genome sequences for non-*H. pylori* species have been generated in the past five years [17–20]. The deposition of this genomic data in the National Center for Biotechnology Information (NCBI) database has encouraged research seeking a deeper understanding of *Helicobacter* species at the genomic level. Therefore, in the present study we aimed to sequence the whole genome of *H. himalayensis*, and to explore the molecular evolution and potential pathogenicity of *H. himalayensis* using genome-wide comparative analyses based on the existing genomes of *Helicobacter* species.

## Results

### General features of *H. himalayensis* genome

The nucleotide sequence of the genome of *H. himalayensis* strain 80(YS1)^T^ was deposited in the NCBI Databases with accession No. CP014991. The final genome assembly of *H. himalayensis* 80(YS1)^T^ contained 103 contigs with a draft genome size of 1,829,936 bp and a genomic GC content of 39.89% (Table 1). The general annotation was performed by the NCBI Prokaryotic Genome Annotation Pipeline (PGAP). It predicted 1769 genes in total, among which 1664 belongs to predicted proteins, 1290 (77.52%) could be assigned to a function with a high level of confidence, and 374 (22.48%) were assigned as hypothetical proteins. The genome contained 39 tRNAs, a pair of 16S rRNAs, a pair of 23S rRNAs, a pair of 5S rRNAs, 3 ncRNAs and one phage intergrase, however, no plasmids or insertion sequence (IS) elements were found. Moreover, a genomic island predicted by a online tool IslandViewer 4, was named HhiG1 in the genome. The genomic island contained multiple genes encoding four restriction endonuclease subunit S, a restriction endonuclease subunit R, a type I restriction-modification system subunit M, a type II toxin-antitoxin system RelE/ParE family toxin, a type II toxin-antitoxin system RelB/DinJ family antitoxin, and site-specific integrase, as well as some hypothetical proteins (See Supplementary Table 1, Additional File 1). HhiG1 comprised 52,677 bp at position 1,738,356 to 1,791,033 in genome .

**Table 1:** General features of the *H. himalayensis*, *H. cinaedi*, *H. hepaticus* and *H. pylori*

Species	***H. himalayensis***	***H. cinaedi***	***H. hepaticus***	***H. pylori***
Strain	80 (YS1)^T^	ATCC BAA 847	ATCC 51449	26,695
Host	Marmota	Human	Rodent	Human
Genome size (bp)	1,829,936	2,240,130	1,799,146	1,667,867
GC content (%)	39.89	38.34	35.93	38.87
Predicted genes	1769	2365	1845	1583
Predicted proteins	1664	2233	1769	1447
Coding area (%)	91	91	93	91
Average gene length (bp)	942	865	881	913
plasmid	None	None	None	None
phages and phage like elements	1 phage intergrase	3 phage like element	3 phage genes	None
IS elements	None	Unknown	None	IS605, IS606
Genomic islands	HHiGI1 (predicted by IslandViewer 4)	Unknown	HHGI1	cag PAI
Regions of deviating GC content	DNA restriction/modification systerm	Unknown	Genomic islets & islands, DNA restriction/ modification system, translation machinery	Genomic islets & islands, DNA-restriction/ modification system, translation machinery

### Phylogenetic relationship and genomic collinearity features of *H. himalayensis* with other *Helicobacter* species

**Fig. 1:**
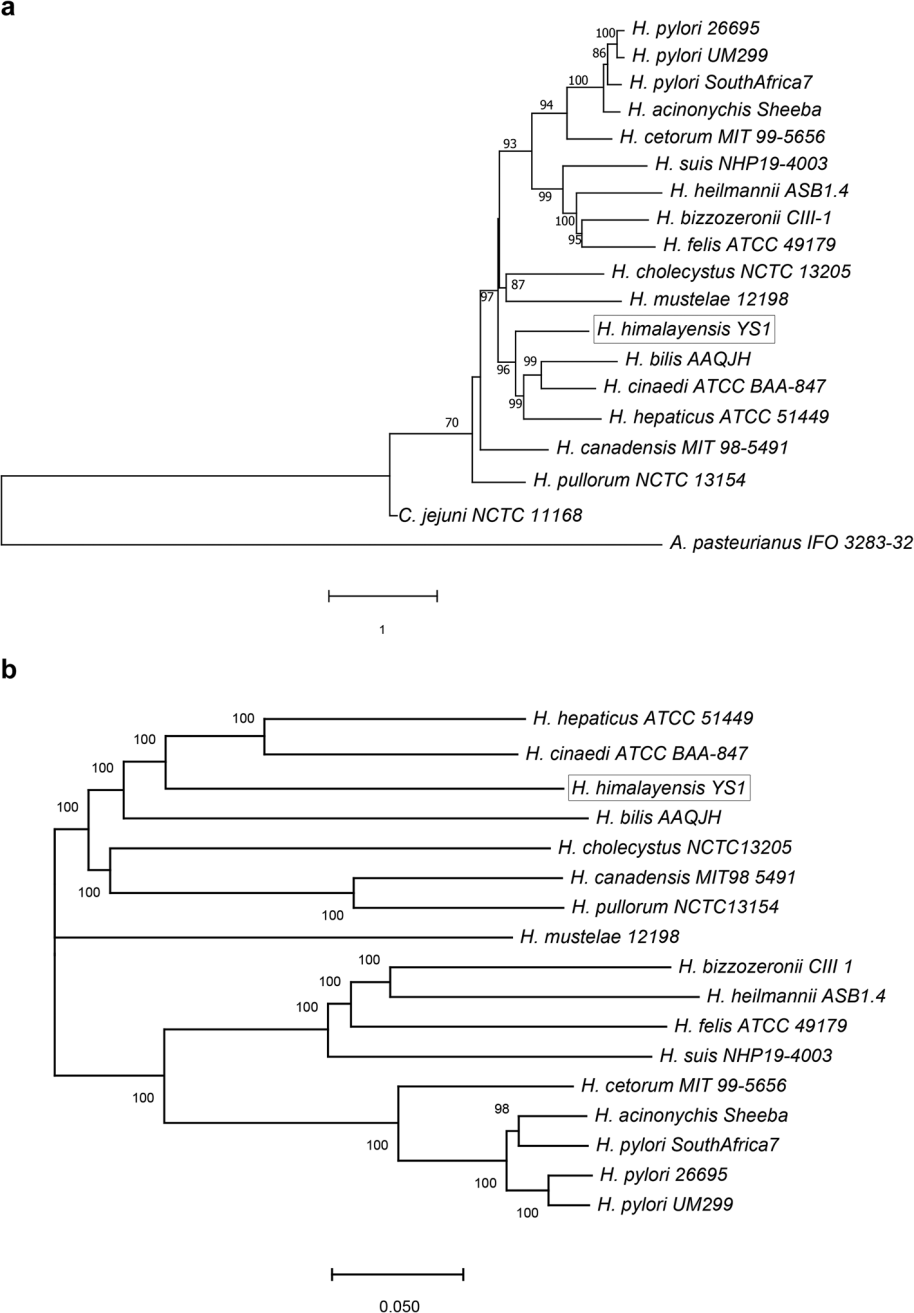
Phylogenetic tree produced by the comparison of the genome sequences of selected bacteria using MEGA X. **a** Phylogenetic tree based on the pan-genomes of the seventeen *Helicobacter* species and two outgroups. *Campylobacter jejuni* NCTC 11168 and *Acetobacter pasteurianus* IFO 3283–32 were used as outgroups. **b** Phylogenetic tree based on the core-genomes of the seventeen *Helicobacter* species without outgroup. The scale indicates the divergence (substitutions/site). The labeled numbers represent the posterior mean of rates on the corresponding branches

Seventeen whole-genome sequences of *Helicobacter* species, including *H. himalayensis*, and two whole-genome sequences of *Campylobacter jejuni* and *Acetobacter pasteurianus* as outgroups, were selected to construct the phylogenetic tree. *H. himalayensis* was in a single clade that was adjacent to *H. cinaedi*, *H. bilis* and *H. hepaticus*. These three *Helicobacter* spp. were in a node that was far away from *H. pylori* (Fig. 1a). Moreover, the phylogenetic relationship between *H. himalayensis* and the selected sixteen *Helicobacter* species, based on the core-genome, were also analyzed (Fig. 1b). The results were consistent with the phylogenetic map based on the *Helicobacter* genome sequences, which indicated that *H. himalayensis* is evolutionarily close to *H. hepaticus* and *H. cinaedi* but not to *H. pylori*. The general genomic features of *H. himalayensis*, *H. cinaedi*, *H. hepaticus*, and *H. pylori* are presented for comparison in Table 1. *H. himalayensis* has a larger genome than that of *H. hepaticus* (1,799,146 bp) and smaller genome than that of *H. cinaedi* (2,240,130 bp) [21]. The genomes of all three *Helicobacter* spp. were larger than that of *H. pylori*. All three *Helicobacter* spp. have phages or phage like elements in their genome, but *H. pylori* has none. By contrast, *H. pylori* has IS elements (IS605 and IS606), whereas *H. himalayensis*, *H. cinaedi,* and *H. hepaticus* do not have.

**Fig. 2:**
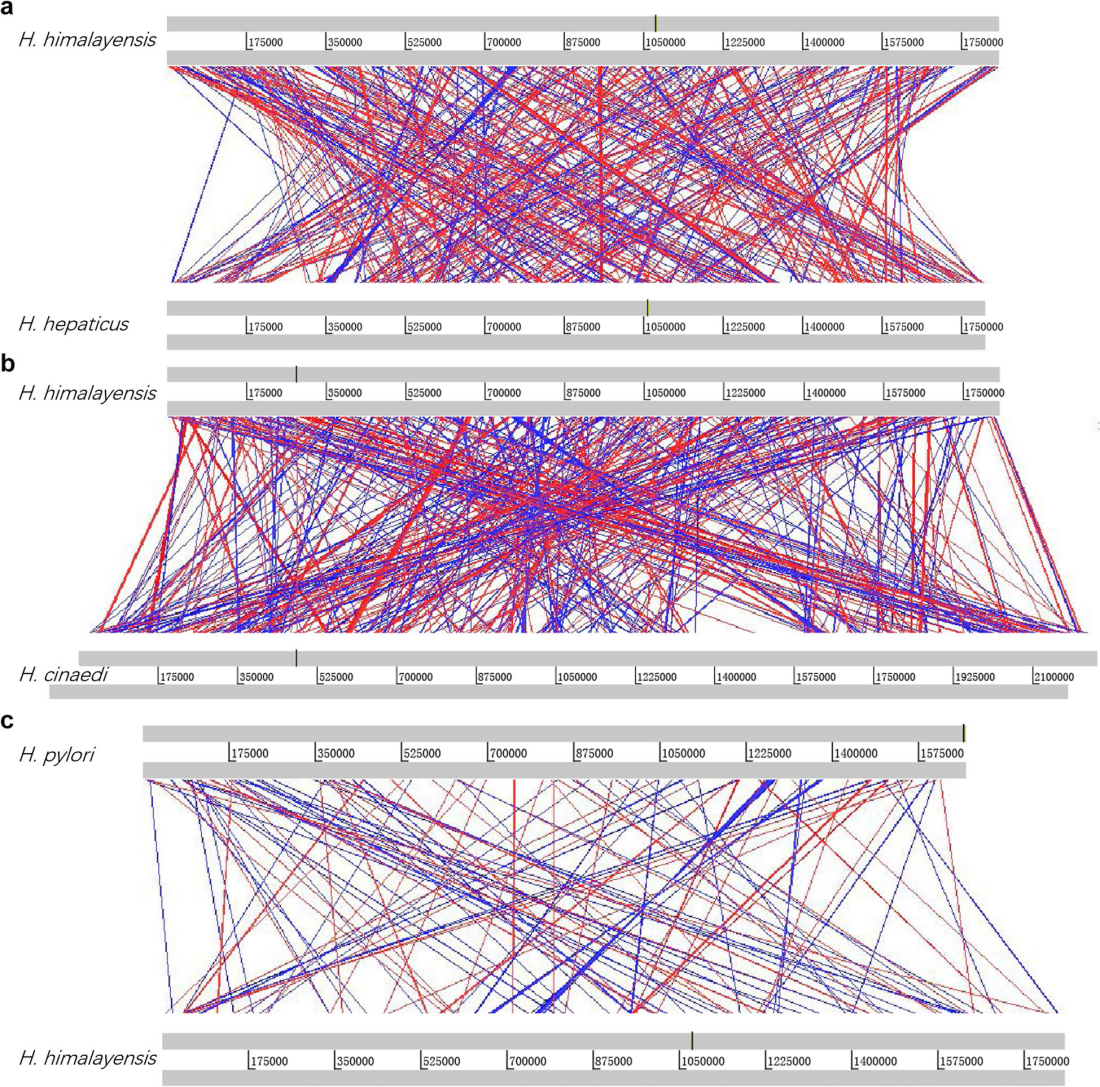
Map of genome collinearity between *H. himalayensis* with *H. hepaticus*, *H. cinaedi*, and *H. pylori*. **a** *H. hepaticus*. **b** *H. cinaedi*. **c** *H. pylori*. A red line indicates that the gene sequence is transcribed in the forward direction, and a blue line indicates that the gene is transcribed in the reverse orientation. The homologous sequences was identified as > 100 bp in length and at least 70% homology

Figure 2 shows a comparison of the gene sequences of *H. himalayensis* with those of *H. cinaedi*, *H. hepaticus*, and *H. pylori* at the whole-genome scale. The results revealed a very high degree of genome collinearity between *H. himalayensis* and *H. hepaticus* (Fig. 2a), as well as between *H. himalayensis* and *H. cinaedi* (Fig. 2b). However, it also revealed a low degree of genome collinearity between *H. himalayensis* and *H. pylori* (Fig. 2c).

### Function analysis of *H. himalayensis*

**Fig. 3:**
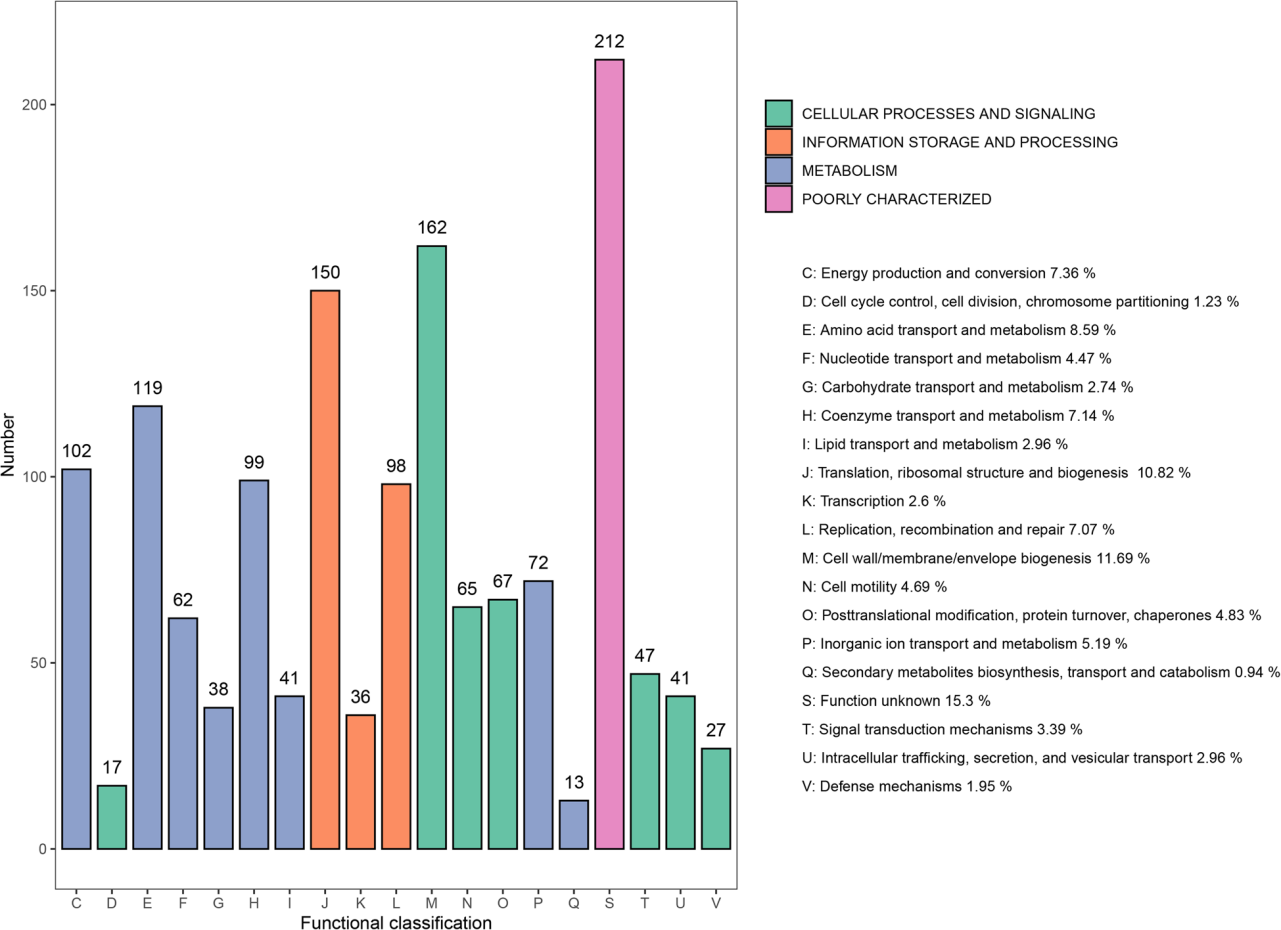
COG functional classification of genes belonging to *H. himalayensis*

**Fig. 4:**
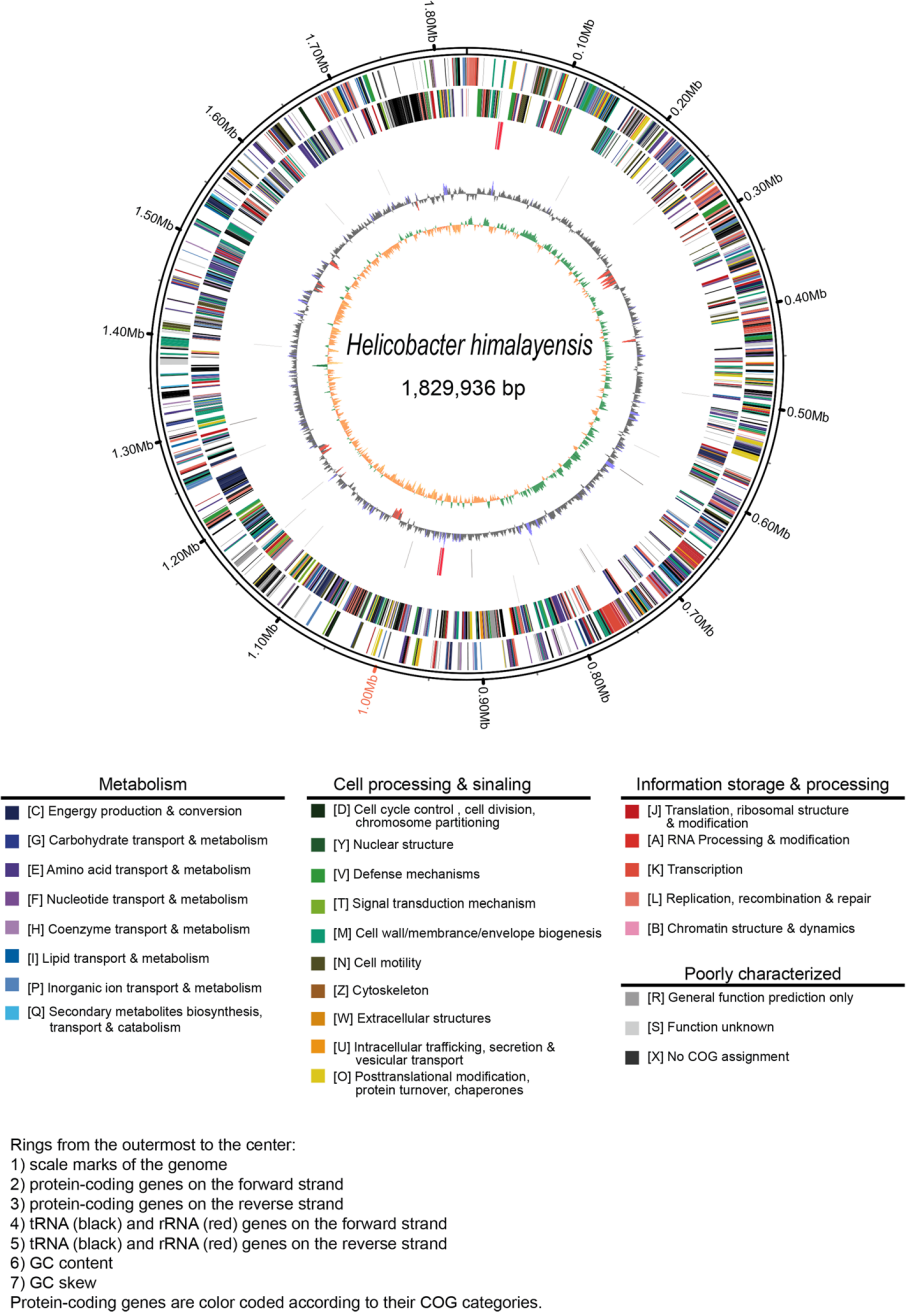
Circular genome atlas of *H. himalayensis*

Of the 1664 predicted proteins, 1184 were clearly assigned to a functional classification with evidence and 202 were poorly functionally characterized using the Clusters of Orthologous Groups (COG) database (Fig. 3). Moreover, there were 105 proteins without any annotations in the COG database and 173 were not in the database. The numbers of genes identified through functional classification in the COG database for “information storage and processing”, “metabolism”, and “cellular processes and signaling” were 284, 546, and 426 respectively. Compared with *H. cinaedi*, *H. hepaticus*, *H. bilis*, and *H. pylori*, *H. himalayensis* has more genes for functions in the “cell wall/membrane/envelope biogenesis” and “coenzyme transport and metabolism” sub-branches, but fewer genes for functions in the “cell cycle control/ cell division/ chromosome partitioning”, “intracellular trafficking/ secretion/ vesicular transport”, “lipid transport and metabolism” and “carbohydrate transport and metabolism” sub-branches (Table 2). The circular genome atlas of *H. himalayensis* integrating different kinds of information is shown in Fig. 4, that including the protein-coding genes, tRNA and rRNA genes, GC content and GC skew information.

**Table 2:** Proportion of genes in COG function clssification for helicobacters

COG function classification	*H.himalayensis*	*H.cinaedi*	*H.hepaticus*	*H.bilis*	*H.pylori*
J: Translation, ribosomal structure and biogenesis	10.82%	8.60%	9.95%	9.53%	11.18%
A: RNA processing and modification	0.00%	0.00%	0.00%	0.06%	0.08%
K: Transcription	2.60%	2.81%	3.16%	3.07%	2.64%
L: Replication, recombination and repair	7.07%	7.75%	5.77%	7.04%	9.32%
D: Cell cycle control, cell division, chromosome partitioning	1.23%	1.57%	1.30%	1.66%	2.17%
V: Defense mechanisms	1.95%	1.85%	1.37%	1.98%	2.56%
T: Signal transduction mechanisms	3.39%	3.88%	3.29%	3.07%	2.41%
M: Cell wall/membrane/envelope biogenesis	11.69%	10.39%	8.99%	10.04%	9.47%
N: Cell motility	4.69%	4.27%	4.46%	4.16%	6.60%
Z: Cytoskeleton	0.00%	0.00%	0.07%	0.00%	0.00%
U: Intracellular trafficking, secretion, and vesicular transport	2.96%	3.76%	3.16%	3.26%	7.14%
O: Posttranslational modification, protein turnover, chaperones	4.83%	4.55%	4.94%	4.61%	4.58%
C: Energy production and conversion	7.36%	7.19%	7.89%	6.33%	6.52%
G: Carbohydrate transport and metabolism	2.74%	3.48%	3.71%	2.88%	2.87%
E: Amino acid transport and metabolism	8.59%	7.75%	8.51%	8.64%	6.52%
F: Nucleotide transport and metabolism	4.47%	4.04%	4.60%	4.29%	5.12%
H: Coenzyme transport and metabolism	7.14%	6.24%	6.31%	5.63%	6.37%
I: Lipid transport and metabolism	2.96%	3.76%	3.64%	3.45%	3.26%
P: Inorganic ion transport and metabolism	5.19%	5.39%	5.28%	5.57%	5.12%
Q: Secondary metabolites biosynthesis, transport and catabolism	0.94%	2.70%	1.72%	1.73%	0.93%
S: Function unknown	15.30%	17.47%	18.53%	19.45%	13.12%

In addition, Forty-two genes were predicted to match virulence factors genes in the VFDB from *Helicobacter* genus, and 83.3% (35/42) of them were associated with flagella motility and bacterial invasion (See Supplementary Table 2, Additional File 2). Other virulence factors included cytolethal distending toxin (*cdtA*, *cdtB*, *cdtC*), lipopolysaccharide Lewis antigens (*futA*), neutrophil activating protein (*napA*), autoinducer-2 production protein (*luxS*), and catalase (*katA*). The *H. himalayensis* genome does not contain a urease and the Cag pathogenic island, like *H. pylori*. However, the genome contained other predicted virulence factors associated with migration, invasion, colonization, and carcinogenesis, which might be pathogenic to the host.

## Discussion

In the present study, the whole genome sequence data of *H. himalayensis*, a new species of *Helicobacter*, were obtained. The *H. himalayensis* genome was compared with those of other *Helicobacter* species deposited in the NCBI database. The results of the comparative genomic analyses not only confirmed phylogenetic position of *H. himalayensis* in relation to other *Helicobacter* species, but also predicted its potential pathogenicity by matching the virulence factor genes in VFDB.

Several smaller stretches of anomalous GC content were identified using the GC mol% content analysis around the genome (Fig. 4). In pathogenic bacteria, this type of anomalous GC content is usually associated with genome islands [22]. For example, the *H. pylori* cag pathogenicity island is intimately associated with increased potential to cause more severe pathology, including cancer and gastric ulcers [23]. There were five fragments with anomalous GC content in the *H. himalayensis* genome, and the predicted genome island HhiG1 was covered by one of the five fragments, which suggested that HhiG1 might be related with the pathogenicity of *H. himalayensis.*

*H. himalayensis* is phylogenetically close to *H. cinaedi* and *H. hepaticus*, based on the whole genome sequence, which is consistent with the previously reported phylogenetic relationship based on the analysis of housekeeping gene analysis [24]. Moreover, the predicted genes of *H. himalayensis* had higher identity to those of *H. cinaedi* and *H. hepaticus* than to those of *H. pylori*, which confirmed the previous result of evolutionary analyses from another perspective. *H. cinaedi* was first isolated from a rectal swab from a homosexual men, while *H. hepaticus* was first isolated from the livers of mice. Both of them are unable to colonize the gastric mucosa, because they do not express a urease to overcome the acidic environment in the stomach. These helicobacters with the term “enterohepatic helicobacters (EHH)” colonize the intestinal mucosa or the liver, and are associated with chronic liver or intestinal inflammation [25]. Recently, many cases of EHH infection in humans were reported, mainly in immunocompromised patients [3]. *H. himalayensis* is closely related to *H. cinaedi* and *H. hepaticus*; therefore, *H. himalayensis* is predicted to have similar pathogenic effects.

Based on the genomic functional analysis of *H. himalayensis*, we found that in addition to the 35 virulence factor genes related to flagellar motility, *H. himalayensis* also has genes encoding CDT, lipopolysaccharide Lewis antigens, neutrophil activating protein, autoinducer-2 production protein, and catalase. These virulence factor genes from the VFDB have been identified in *H. hepaticus*, *H. pylori*, or other bacteria in experiments [26]. CDT is a typical virulence factor, having DNase I activity that induces DNA double-strand breaks, apoptosis, and G2/M cell cycle arrest in cultured mammalian cells. In vivo, CDT is also associated with carcinogenesis [27, 28]. The Lewis antigen (encoded by *futA*) suppresses the immune response to the bacteria permits it to adhere to the gastric mucosa, or has a role in certain aspects of autoimmunity [29]. Neutrophil activating protein (encoded by *napA*) could promote the production of reactive oxygen radicals in *H. pylori* or the adhesion of human neutrophils to endothelial cells [30, 31]. Autoinducer-2, encoded by *luxS*, is produced and detected by a wide variety of bacteria, and is presumed to facilitate interspecies communications [32]. *katA* is a gene coding catalase, which protects against reactive oxygen species damage [33]. It is hypothesized that these orthologous genes in the *H. himalayensis* genome have similar effects and may also confer potential pathogenicity on the host. Till now, no animal experiment has been done with *H. himalayensis* infection, but the result of histopathological examination for the gastrointestinal mucosa of *M. himalayana* where *H. himalayensis* isolated showed obvious lesions, while the gastrointestinal mucosa of *M. himalayana* without *H. himalayensis* isolated had no lesions (See Supplementary Figure 1, Additional File 3). Therefore, whether *H. himalayensis* containing these virulence factor genes is a pathogen to host, should be confirmed by further experimental studies.

## Conclusions

The present study described the characteristic of the *H. himalayensis* genome, analyzed its phylogenetic relationship with other helicobacters, and highlighted the potential pathogenicity of 80(YS1)^T^ to the host. Further research into the pathogenesis of this potentially pathogenic bacterium is necessary and might help to manage diseases caused by *H. himalayensis*.

## Methods

### Bacterial strain

*H. himalayensis sp*. nov strain 80(YS1)^T^ (identical to CGMCC1.12864 and DSM28742) was used in this study. It was cultured as previously described, with some modifications [24]. Briefly, the stored strain was revived and cultured on brain heart infusion agar plates containing 8% (v/v) horse blood, 2.5 μg/mL trimethoprim, 10 μg/mL vancomycin, and 1.25 U/mL polymyxin B, under a hypoxic atmosphere (5% CO_2_, 5% H_2_, 0.5% O_2_, and 90% N_2_) at 35 °C for 3–5 days.

### Genome DNA extraction and whole genome sequencing

A Wizard® Genomic DNA Purification kit (Promega, Madison, WI, USA) was used to isolate the genomic DNA from collected cells of strain 80(YS1)^T^, according to the manufacturer’s instructions. Sequencing and library construction were carried out using the Hiseq2000 90 bp paired end sequencing platform (Illumina, San Diego, CA, USA. In total, 273,256 polymerase reads (242 bp/reads) were generated with 33.1× theoretical coverage. After filtering out all low quality reads, 266,891 reads (97.7%) were assembled into 103 contigs using 454/Roche Newbler (Roche Molecular Systems Inc., Branchburg, NJ, USA). All the contigs were sorted using the genomes of *H. pylori* 26695 and *H. cinaedi* ATCC BAA847, and verified by sequencing using an ABI BigDye Terminator V3.1 Kit and ABI 3730 sequencer (Applied Biosystems, Foster City, CA, USA). Conventional Sanger sequencing of polymerase chain reaction (PCR) fragments, based on brute force PCR, was used to for gap filling among the contigs and scaffolds to complete the genome.

### Comparative genomics analysis

The assembled genome of strain 80(YS1)^T^ was compared with the available *Helicobacter spp.* genomes deposited in the NCBI Database. Sixteen *Helicobacter spp.* and two outgroup stains (*Campylobacter jejuni* and *Acetobacter pasteurianus*) were selected, and the whole genomes were download from NCBI Database. For pan-genome analysis, SNP sequence from the selected genomes were generated using KSNP3 (version 3.0) [34], and analysed using MEGA X [35] to construct the phylogenetic trees by Neighbor-Joining algorithm with 1000 bootstraps. For core-genome analysis, genes present in each selected Helicobacter genome were classified as core. The core-genome compared files were generated using roary (version 3.12.0) with a BLAST cutoff of 70% identity of gene (−i 70). MEGA X was used to construct the phylogenetic trees by Neighbor-Joining algorithm with 1000 bootstraps based on the core-genome. Collinearity analysis of *H. hemalayensis* with other three helicobacter species were performed using BLASTn. To identify homologous sequences (> 100 bp in length and at least 70% homology), 80(YS1)^T^ genomic contigs were aligned with those of three other *Helicobacter* species, using BLASTn as implemented in NCBI BLAST+ v. 2.2.8 [36]. Progressive Mauve, with match seed weight = 15, minimum Locally Collinear Block weight = 45, minimum island size = 50, maximum backbone gap size = 50, and a minimum backbone size = 50 [37] was used to compare the genomes of the *Helicobacter* spp. using multiple sequence alignment. CIRCOS v. 0.64 [38] was used to construct a visual representation of the links between homologous regions of *H. himalayensis sp*. nov strain 80(YS1)^T^ and those of three other *Helicobacter* species.

### Function analysis of *H. himalayensis*

The identified *H. himalayensis* proteins were assigned and annotated to the database of COG by eggnog-mapper (version 2) [39]. COG classification was conducted by using DIAMOND v0.8.37.99 (−evalue 1e-5) [40]. The results of the COG classification were visualized using R (version 3.6.2). The genomic island of *H. himalayensis* was predicted by an online tool IslandViewer 4 [41] (http://www.pathogenomics.sfu.ca/islandviewer/upload/). The virulence factors of *H. himalayensis* were searched in the virulence factors database (VFDB) using the integrated and automatic pipeline, Vfanalyzer [42] (http://www.mgc.ac.cn/cgi-bin/VFs/v5/main.cgi?JobID=May_28-1427107863).

## Supplementary Information

**Additional file 1: Supplementary Table 1.** Results of genomic island predicted by Island Viewer 4.

**Additional file 2: Supplementary Table 2.** Virulence factors genes present in genome of *H.himalayensis.*

**Additional file 3: Supplementary Figure 1.** Samples for histological examination. **a** Image and **c** histological examination of the intestinal mucosa of *Marmota himalayana* without *H. himalayensis* isolated (hematoxylin-eosin, original magnification × 100). A black arrow indicates normal histoarchitecture of the intestinal mucosa. **b** Image and **d** pathological examination of the intestinal mucosa of *Marmota himalayana* where *H. himalayensis* was isolated (hematoxylin-eosin, original magnification × 100). A black arrow indicates necrosis area and/or lesion area of the intestinal mucosa.

## Data Availability

The datasets analysed during the current study are available in the NCBI repository, accession numbers: NZ_CP014991 for *H. himalayensis* strain 80 (YS1)^T^, complete genome; NC_008229 for *H. acinonychis* Sheeba, complete genome; NZ_CP019645 for *H. bilis* strain AAQJH, complete genome; NC_015674 for *H. bizzozeronii* CIII-1, complete genome; NZ_CM000776 for *H. canadensis* MIT 98–5491; NC_017735 for *H. cetorum* MIT 99–5656, complete genome; NZ_LR134518 for *H. cholecystus* strain NCTC13205, complete genome; AP012492 for *H. cinaedi* ATCC BAA-847, complete genome; NC_014810 for *H. felis* ATCC 49179, complete genome; NC_019674 for *H. heilmannii* ASB1.4, complete genome; AE017125 for *H. hepaticus* ATCC 51449, complete genome; NC_013949 for *H. mustelae* 12198, complete genome; NZ_LR134509 for *H. pullorum* NCTC13154, complete genome; CP003904 for *H. pylori* 26695, complete genome; NC_017361 for *H. pylori* SouthAfrica7, complete genome; CP005491 for *H. pylori* UM299, complete genome; AP023039 for *H. suis* NHP19–4003, complete genome; NC_017111 for *Acetobacter pasteurianus* IFO 3283–32, complete genome; NC_002163 for *Campylobacter jejuni* NCTC 11168, complete genome.

## Abbreviations

NCBI: National Center for Biotechnology Information
CDT: Cytolethal Distending Toxin
IS: Insertion Sequence
COG: Clusters of Orthologous Groups
VFDB: Virulence factors database
EHH: Enterohepatic helicobacters
