# Supplementary material for: Genomic analysis of Helicobacter himalayensis sp. nov. isolated from Marmota himalayana
Source: BMC Genomics. 2020 Nov 23;21:826. doi: 10.1186/s12864-020-07245-y (PMC7685656; doi:10.1186/s12864-020-07245-y)
Supplement: Supplementary file 2 — Additional file 2: Supplementary Table 2. Virulence factors genes present in genome of H.himalayensis. [file 12864_2020_7245_MOESM2_ESM.docx]

**Supplementary Table 2.** Virulence factors genes present in genome of *H.himalayensis*

| **NO.** | ***H.himalayensis*** | **Start position** | **End position** | **Matched Genes in VFDB** | **Functional Category** |
| --- | --- | --- | --- | --- | --- |
| 1 | orf02534 | 1663406 | 1664446 | *futA* | Lipopolysaccharide Lewis antigens |
| 2 | orf01218 | 784326 | 784763 | *napA* | Neutrophil-activating protein |
| 3 | orf02425 | 1583551 | 1585140 | *flaA* | Flagella |
| 4 | orf00598 | 401562 | 403106 | *flaB* |  |
| 5 | orf00681 | 462307 | 462744 | *flgB* |  |
| 6 | orf00680 | 461797 | 462291 | *flgC* |  |
| 7 | orf02499 | 1634505 | 1635326 | *flgD* |  |
| 8 | orf02145 | 1408147 | 1410300 | *flgE_1* |  |
| 9 | orf02500 | 1635323 | 1637533 | *flgE_2* |  |
| 10 | orf02739 | 1795654 | 1796481 | *flgG_1* |  |
| 11 | orf00471 | 324326 | 325114 | *flgG_2* |  |
| 12 | orf02024 | 1320743 | 1321456 | *flgH* |  |
| 13 | orf00288 | 201462 | 202481 | *flgI* |  |
| 14 | orf02444 | 1593678 | 1595498 | *flgK* |  |
| 15 | orf01560 | 1025118 | 1027682 | *flgL* |  |
| 16 | orf02162 | 1421266 | 1423491 | *flhA* |  |
| 17 | orf00023 | 22094 | 23218 | *flhB_1* |  |
| 18 | orf00273 | 190017 | 190313 | *flhB_2* |  |
| 19 | orf00083 | 69423 | 70889 | *flhF* |  |
| 20 | orf00086 | 72205 | 72900 | *fliA* |  |
| 21 | orf00095 | 79283 | 81337 | *fliD* |  |
| 22 | orf00679 | 461443 | 461766 | *fliE* |  |
| 23 | orf01177 | 748103 | 749812 | *fliF* |  |
| 24 | orf01179 | 749822 | 750853 | *fliG* |  |
| 25 | orf01180 | 750840 | 751646 | *fliH* |  |
| 26 | orf00699 | 471619 | 472968 | *fliI* |  |
| 27 | orf00549 | 369176 | 369733 | *fliL* |  |
| 28 | orf00087 | 72893 | 73957 | *fliM* |  |
| 29 | orf01627 | 1066642 | 1066995 | *fliN* |  |
| 30 | orf02483 | 1624205 | 1625164 | *fliP* |  |
| 31 | orf00740 | 496722 | 496973 | *fliQ* |  |
| 32 | orf02283 | 1493016 | 1493786 | *fliR* |  |
| 33 | orf00096 | 81347 | 81736 | *fliS* |  |
| 34 | orf00088 | 73963 | 74793 | *fliY* |  |
| 35 | orf02538 | 1666760 | 1667500 | *motA* |  |
| 36 | orf02537 | 1666001 | 1666750 | *motB* |  |
| 37 | orf01901 | 1231967 | 1234318 | *pflA* |  |
| 38 | orf02374 | 1555976 | 1556695 | *cdtA* | Cytolethal distending toxin |
| 39 | orf02373 | 1555142 | 1555966 | *cdtB* |  |
| 40 | orf02372 | 1554575 | 1555129 | *cdtC* |  |
| 41 | orf00570 | 384321 | 384806 | *luxS* | Autoinducer-2 |
| 42 | orf02151 | 1414270 | 1415703 | *katA* | Catalase |
