# Supplementary material for: Genomic analysis of Helicobacter himalayensis sp. nov. isolated from Marmota himalayana
Source: BMC Genomics. 2020 Nov 23;21:826. doi: 10.1186/s12864-020-07245-y (PMC7685656; doi:10.1186/s12864-020-07245-y)
Supplement: Supplementary file 3 — Additional file 3: Supplementary Figure 1. Samples for histological examination. a Image and c histological examination of the intestinal mucosa of Marmota himalayana without H. himalayensis isolated (hematoxylin-eosin, original magnification × 100). A black arrow indicates normal histoarchitecture of the intestinal mucosa. b Image and d pathological examination of the intestinal mucosa of Marmota himalayana where H. himalayensis was isolated (hematoxylin-eosin, original magnification × 100). A black arrow indicates necrosis area and/or lesion area of the intestinal mucosa. [file 12864_2020_7245_MOESM3_ESM.pptx]

## Slide 1
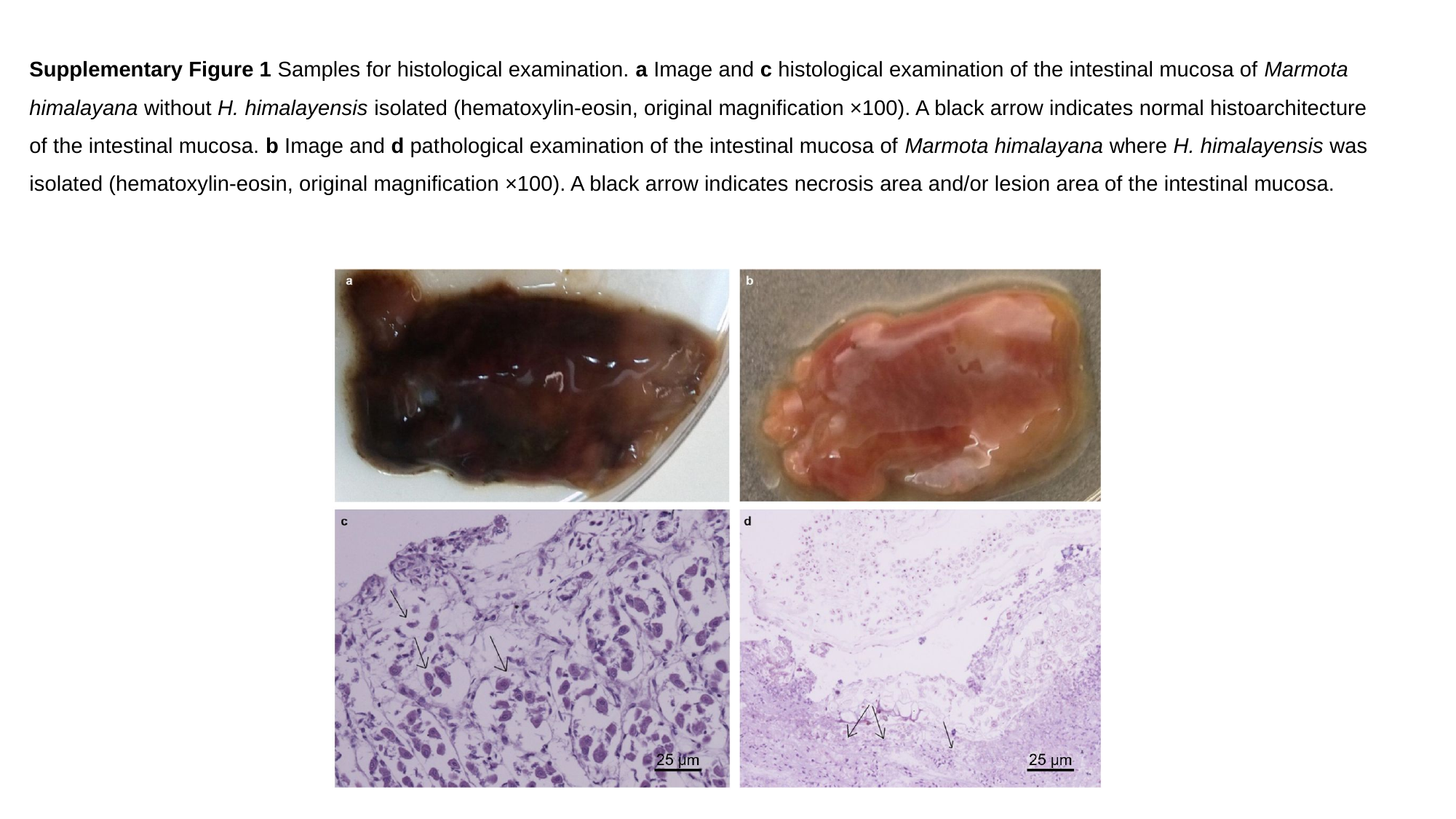

# Supplementary Figure 1 Samples for histological examination. a Image and c histological examination of the intestinal mucosa of Marmota himalayana without H. himalayensis isolated (hematoxylin-eosin, original magnification ×100). A black arrow indicates normal histoarchitecture of the intestinal mucosa. b Image and d pathological examination of the intestinal mucosa of Marmota himalayana where H. himalayensis was isolated (hematoxylin-eosin, original magnification ×100). A black arrow indicates necrosis area and/or lesion area of the intestinal mucosa.
